# A person‐centred approach to enhance the long‐term health and wellbeing of people living with HIV in Europe

**DOI:** 10.1002/jia2.26117

**Published:** 2023-07-06

**Authors:** Jeffrey V. Lazarus, Mario Cascio, Jane Anderson, Sini Pasanen, Richard Harding

**Affiliations:** ^1^ Barcelona Institute for Global Health (ISGlobal) Hospital Clínic University of Barcelona Barcelona Spain; ^2^ CUNY Graduate School of Public Health and Health Policy (CUNY SPH) New York New York USA; ^3^ Faculty of Medicine and Health Sciences University of Barcelona Barcelona Spain; ^4^ European AIDS Treatment Group Brussels Belgium; ^5^ Homerton Healthcare NHS Foundation Trust London London UK; ^6^ Positiiviset HivFinland and AIDS Action Europe Helsinki Finland; ^7^ Florence Nightingale Faculty of Nursing Midwifery and Palliative Care Cicely Saunders Institute King's College London London UK

1

Person‐centred care is a critical attribute of high‐quality healthcare, promoting quality of life, improving an individual's interaction with the health system and valuing people's social networks [1]. Lifesaving antiretroviral therapy is now increasingly widely available around the world, although not all countries have reached their coverage targets related to prevention, diagnosis and linkage to care. As a consequence of the enormous progress made, most people living with HIV are able to grow older rather than dying prematurely [2]. Therefore, it is essential that health systems respond to the changing needs of an ageing population living with HIV, who have comparatively higher multimorbidity (both physical and mental) and experience persistent stigma [3, 4].

To achieve this, person‐centred care services for people living with HIV must focus on long‐term wellbeing by monitoring and managing multimorbidity, health‐related quality of life (HRQoL), stigma and discrimination [5]. In line with WHO's global strategy on HIV for 2022–2030 [2] and UNAIDS' targets set in 2021 [6], a people‐centred approach is essential to allow for this transformation of health systems. Health services must offer an integrated response to the evolving health needs and choices of people living with HIV—and this is still not the case in most settings, including high‐income countries in Europe [7].

The crisis with health systems during 2020 and the worsening of health outcomes during the COVID‐19 pandemic [8] demonstrated the importance of coordination among European countries to protect population health across national borders, especially among the most vulnerable. Against the backdrop of the pandemic, in 2020, the European Union put forward a proposal for a European Health Union [9]. Ambitious policy opportunities were identified, from a proposal for a European Health Data Space, to reinforcing the European Centre for Disease Prevention and Control mandate and pushing forward the European Care Strategy [10]. Person‐centricity is at the corej of these health system initiatives, paving the way for personalized healthcare delivery. These initiatives are of particular relevance for people living with HIV and their healthcare services given their multidimensional health and social care needs.

A people‐centred health system is organized around what is important to people living with HIV, their needs and choices, rather than focusing on individual diseases [5, 11]; it “consciously adopts the perspectives of individuals, families and communities (…), sees them as participants and beneficiaries of trusted health systems that respond (…) in humane and holistic ways” and “acknowledges the experiences and perspectives of health‐care providers that may enable or prevent the delivery of [these health services]” [12]. In the context of HIV, people living with HIV must be empowered and supported to make decisions about the degree of self‐management that they are capable of and willing to assume in line with their age, gender, socio‐economic status and support network.

A key objective of a people‐centred approach is to ensure that the views, needs and wellbeing of people living with HIV are represented in policy initiatives by prioritizing the enhancement of their long‐term health, HRQoL and overall wellbeing, from diagnosis until the end of life [5]. With this goal in mind, in 2021 and 2022, over 60 multidisciplinary HIV organizations and experts from the HIV Outcomes initiative co‐developed a set of 27 policy asks and recommendations for European health systems and authorities [13]. Qualitative input was collected with a representation of people living with HIV, clinicians, academics, public health professionals, policymakers and industry representatives. Expert interviews were conducted via a questionnaire and at seven workshops. Entitled “Enhancing long‐term health and well‐being among people living with HIV,” the policy asks were grouped into four areas where policy action is most urgent to improve wellbeing: (1) comorbidity prevention, treatment and management‐including mental health; (2) ageing with HIV; (3) patient‐reported outcomes measures and national monitoring of HRQoL; and (4) combatting stigma and discrimination. For each area, specific, implementable and translatable recommendations were made for HIV clinics/care providers, national and regional authorities and European Union health policymakers. Priority recommendations are highlighted in Table 1 [13].

**Table 1:** Summary of policy recommendations for each area of action in the European context

Areas of action	Priority recommendations	
**Comorbidity prevention, treatment and management**	**HIV clinics/care providers**	Implement routine screening for all relevant comorbidities based on individual characteristics and needs, in line with national and international guidelines, using short, easy‐to‐answer and validated screening instruments. Capture relevant data using electronic health records as a tool to support integrated, personalized care. Inclusion of management and referral protocols for the range of person‐centred problems identified. Involve peers or community members to support with prevention, screening, treatment and management of comorbidities.

	**National and regional authorities**	Develop or update a monitoring and evaluation framework for HIV care, incorporating indicators on comorbidities, leading causes of mortality and hospitalization and patient‐reported outcomes, including HRQoL. Integrate the framework into the national HIV strategy and ensure funding for its implementation. Include other communicable diseases and HRQoL as core elements of national HIV strategies.

	**European Union**	Expand the mandate of the European Commission's Steering Group on Health Promotion, Disease Prevention and Management of Non‐Communicable Diseases to initiate work programmes on communicable diseases, such as HIV, including prevention, diagnosis and coordinated management of comorbidities. Ensure sustainable funding for cohort studies to provide information on the long‐term health of people living with HIV, including comorbidities and HRQoL.
**Ageing with HIV**	**HIV clinics/care providers**	Deliver specialized, integrated healthcare and social services focused on the needs of older adults living with HIV: frailty and other geriatric syndromes, disability, age‐related comorbidities, as well as mental and sexual health and active ageing.

	**National and regional authorities**	Develop and implement training programmes for carers, in particular those working in retirement homes, focused on the specific health and wellbeing needs of older adults living with HIV, including mental and sexual health and active ageing.

	**European Union**	Provide funding for pilot studies on models of HIV care that employ or develop frameworks for healthy ageing, frailty, functional ability and other dimensions of health that are relevant to people living with HIV, using HRQoL as a key outcome measure.
**PROMs and HRQoL**	**HIV clinics/care providers**	Integrate PROMs into clinical practice, which can then be used for shared decision‐making with those living with HIV, to tailor interventions to meet the needs and preferences of individuals and for monitoring of health outcomes.

	**National and regional authorities**	Implement methodologically robust annual surveys of people living with HIV to collect and document data on HRQoL and on experiences of stigma and discrimination in healthcare settings.

	**European Union**	Allocate funding for the inclusion of HIV within the OECD Paris Initiative to provide standardized, comparable data on person‐reported outcomes and person‐reported experiences across countries.
**Stigma and discrimination**	**HIV clinics/care providers**	Offer peer‐to‐peer and community‐based interventions that address stigma and discrimination experienced by people living with HIV, including a focus on the fact that an undetectable viral load means an untransmissible virus.

	**National and regional authorities**	Design and implement interventions that can strengthen empathy towards people living with HIV among healthcare staff, disseminating the U = U message in order to decrease stigma and discrimination in and outside of healthcare settings.

	**European Union**	Ensure that any future EU mental health strategy includes a focus on people experiencing stigma and discrimination, including people living with HIV specifically.

Abbreviations: EU, European Union; HRQoL, health‐related quality of life; OECD, Organization for Economic Cooperation and Development; PROMs, patient‐reported outcome measures; U = U, Undetectable = Untransmittable.

Although focusing on monitoring HRQoL may be challenging for countries or health systems with fewer resources available, people living with HIV experience a greater overall burden of multimorbidity in comparison with the general population and reduced HRQoL across all domains. Therefore, actions that can benefit both individual wellbeing and health system costs need to be adopted. This should be in tandem with efforts to increase the numbers of people diagnosed, linked to care and virally suppressed [14]. A particular focus should be placed on reaching people who are diagnosed late as they are at high risk of clinical progression and poor outcomes. Currently, 53% of people newly diagnosed with HIV in the WHO European region are diagnosed late, of whom 51% are aged >50 [15]. We encourage the adoption of these measures by all European Union member states by the end of 2023. The aforementioned policy recommendations are tailored to tackle the shortcomings in healthcare settings for people living with HIV in Europe. Different priority areas of action also may be considered for other regions of the world.

## COMPETING INTERESTS

JVL reports grants and speaker fees from AbbVie, Gilead Sciences, MSD and Roche Diagnostics to his institution, speaker fees from Intercept, Janssen, Novo Nordisk and ViiV and consulting fess from Novavax, outside of the submitted work. JA reports consultancy and speaker fees from Gilead Sciences and speaker fees from ViiV Healthcare, outside of the submitted work. MC, SP and RH have no competing interest to declare.

## AUTHORS’ CONTRIBUTIONS

JVL conceived of the paper. MC, JA, SP and RH reviewed the first full draft of the article. All authors were involved in subsequent revisions and approved the final version for submission.
